# Comparison of Bone Mineral Density in US Adults With Diabetes, Prediabetes and Normoglycemia From 2005 to 2018

**DOI:** 10.3389/fendo.2022.890053

**Published:** 2022-05-30

**Authors:** Jing Yuan, Pu Jia, Jian-Bo Zhou

**Affiliations:** ^1^ Department of Endocrinology, Beijing Tongren Hospital, Capital Medical University, Beijing, China; ^2^ Department of Orthopedics, Beijing Friendship Hospital, Capital Medical University, Beijing, China

**Keywords:** diabetes, prediabetes, bone mineral density, dual-energy x-ray absorptiometry, National Health and Nutrition Examination Survey

## Abstract

**Background:**

Accumulating evidence has shown that diabetes has an impact on bone metabolism with conflicting results. Furthermore, little is known about the relationship of prediabetes with bone mineral density (BMD). Therefore, we explored the association between BMD and glucometabolic status in adults in the US.

**Methods:**

In this cross-sectional study, we extracted and analyzed data from the National Health and Nutrition Examination Survey (NHANES) from 2005 to 2018. A total of 14610 subjects aged 40 ≥ years diagnosed with diabetes, prediabetes, or normal glucose regulation (NGR) and had available data on BMD were eligible.

**Results:**

The prevalence of prediabetes and diabetes in US adults aged 40 ≥ years were 39.2% and 26.4%, respectively. After multivariable adjustment, we found an increasing trend of BMD at the total hip, femoral neck, and lumbar spine with glucometabolic conditions from NGR and prediabetes to diabetes in adults aged ≥ 40 years in the US. This trend was more prominent in women than that in men. Fasting plasma glucose (FPG) and HbA1c levels were also positively correlated with BMD.

**Conclusions:**

Glucometabolic conditions were significantly associated with BMD values in US adults.

## Introduction

Diabetes mellitus (DM) is a chronic metabolic disorder characterized by insufficient insulin production, endogenous insulin resistance, or both (1, 2), resulting in hyperglycemia. This results in an increased risk of microvascular and macrovascular complications, which significantly impact an individual’s life expectancy and quality of life (3, 4). According to the International Diabetes Federation, approximately 537 million adults worldwide have diabetes, which is expected to rise to 783 million by 2045 (5). The high prevalence of diabetes and its associated complications impose an overwhelming medical and economic burden and is emerging as a severe public health issue.

Substantial evidence has linked diabetes with disorders of bone metabolism and skeletal fragility, which have been suggested to be novel complications of long-term exposure to an uncontrolled blood glucose environment (6, 7). The mechanisms underlying the deterioration of bone quality in diabetes are complex and poorly documented. Several factors, including oxidative stress, accumulation of advanced glycation end products, inflammatory cytokines, muscle-derived hormones, incretins, hydrogen sulfide production, and cortisol secretion, negatively affect bone strength (7, 8).

Prediabetes, defined as the presence of impaired fasting glucose (IFG), impaired glucose tolerance (IGT), and/or mildly elevated hemoglobin A1c (HbA1c), precedes the onset of diabetes. Emerging insights into macrovascular disorders such as cardiovascular disease, stroke, and peripheral vascular disease are relevant to prediabetes (9–12). Current evidence suggests an association between prediabetes and the prevalence of microvascular complications (13). However, few studies with adequate sample sizes have investigated the influence of prediabetes on skeletal health, and there is controversy in the results with increased (14), decreased (15), or similar (16) values of bone mass density (BMD) when compared with normoglycemic individuals. Furthermore, little is known about the trend that exists between BMD and blood glucose status.

In this study, we investigated BMD trends in US adults aged ≥ 40 years with diabetes, prediabetes, and normal glucose regulation (NGR) using nationally representative sample data from the National Health and Nutrition Examination Survey (NHANES) from 2005 to 2018.

## Methods

### Data Source

The data for this cross-sectional study were obtained from the NHANES, a survey conducted by the National Center for Health Statistics (NCHS) of the Centers for Disease Control and Prevention (CDC) to assess the health and nutritional status of a nationally representative sample of the US civilian population using a multistage, stratified, clustered probability design. Questionnaire interviews, laboratory tests, and physical examinations were included in the survey. The continuous datasets of the NHANES 2005–2006, 2007–2008, 2009–2010, 2013–2014 and 2017–2018 were combined in this study. NHANES 2011–2012, 2015–2016 were excluded because of the lack of data on the lumbar spine and femur BMD during these periods. The NHANES study protocol was approved by the NCHS Ethics Review Board, and written informed consent was obtained from all participants (17, 18).

### Study Participants

Dual-energy X-ray absorptiometry (DXA) scans were only administered to eligible survey participants aged ≥ 40 years in the 2013–2014 cycle. This study population was limited to subjects aged ≥40 years who were diagnosed with diabetes, prediabetes, or NGR with available data on BMD. Among the total 50463 participants for the 5-cycle survey, after the exclusion of 17445 subjects without glucose status (n=33018) and 10250 without valid BMD data (n=22768), 14610 subjects aged ≥40 years were enrolled in our study for final analysis.

### Definition of Glucose Status

Diabetes status was confirmed when subjects had a positive response to the question, “Have you ever been told by a doctor that you have diabetes?”. Furthermore, the diagnosis of diabetes was also based on American Diabetes Association (ADA) criteria (19), which is fasting plasma glucose (FPG) ≥ 7.0 mmol/l or 2-h plasma glucose (2h-PG) after oral glucose tolerance test (OGTT) ≥ 11.0 mmol/l or HbA1c ≥ 6.5%. Participants were diagnosed with prediabetes who answered “yes” to the question “Have you ever been told by a doctor that you have prediabetes?” or had FPG ≥ 6.1, < 7.0 mmol/L, or 2h-PG ≥ 7.8, < 11.0 mmol/L or HbA1c ≥ 5.7%, < 6.5%.

### BMD Measurements

BMD of the total femur and lumbar spine was assessed using DXA scans with Hologic QDR-4500A fan-beam densitometers (Hologic, Inc., Bedford, Massachusetts, USA) in the NHANES mobile examination center (MEC). The detailed examination protocol for DXA is described on the NHANES website. BMD values of the total hip, femoral neck, trochanter, intertrochanter, Ward’s triangle, and vertebrae L1–L4 were obtained from the DXA profiles. Based on the osteoporosis diagnostic criteria of the World Health Organization (WHO) (20), BMD values of the hip, femoral neck and vertebrae L1-4 were included in the current study.

### Study Variables

Demographic information, including age, gender (male or female), race/ethnicity (non-Hispanic white, non-Hispanic black, Mexican American, other races), educational level (below high school, high school or equivalent, above high school), marital status (married/living with a partner, widowed/divorced/separated, and never married), ratio of family income to poverty (PIR: ≤1, 1 < to ≤ 3, > 3), smoking at least 100 cigarettes in life (yes/no), and vigorous recreational activities (yes/no) were collected from the NHANES database. Body mass index (BMI) was calculated as the weight (kg) divided by the squared height (m^2^). The systolic blood pressure (SBP) and diastolic blood pressure (DBP) were measured. Data on serum creatinine (sCr), uric acid (UA), total cholesterol (TC), triglyceride (TG), alkaline phosphatase (ALP), serum calcium, and serum phosphorus were also extracted from the database. The detailed process of the data collection was obtained from the NHANES website.

### Statistical Analysis

Based on the NHANES analytical guidelines, sampling weights were used for all analyses to interpret the complicated NHANES survey design. Ten-year weights were calculated for the 2005–2010, 2013–2014 and 2017–2018 estimates by multiplying the 2-year weights by one-fifth. Continuous variables are expressed as mean ± standard deviation, and comparisons between the three groups were made using one-way ANOVA. Categorical measures were expressed as percentages, and a Chi-square test was used for analysis. Multivariable linear regression analysis was used to evaluate the association between glucose status and BMD in the three models. The regression coefficient (β) and 95% confidence interval (95% CI) were estimated. Model 1 was unadjusted. Model 2 was adjusted for age, sex, and race. Model 3 was adjusted for model 2 adjustments plus BMI, education level, marital status, ratio of family income to poverty, smoking at least 100 cigarettes in life, vigorous recreational activities, ALP, total calcium, uric acid, creatinine, and cholesterol. SPSS software (version 17.0; SPSS Inc., Chicago, IL, US), STATA 15.0 software (StataCorp LLC, College Station, TX, USA) were used for statistical analyses. A two-sided P value < 0.05 is considered statistically significant.

## Results

### General Characteristics of All Participants

Of the 14610 participants, 5730 (39.2%) had prediabetes and 3864 (26.4%) had diabetes, accounting for 65.6% of the entire cohort. Participants with diabetes and prediabetes differed from those with NGR in several demographic and biochemical indices (**Table 1**). There were significantly more elderly and fewer non-Hispanic Whites in the prediabetes and diabetes groups than in the NGR group. Compared with NGR, subjects with diabetes and prediabetes appeared to have older age, lower education level, lower PIR, more smoking habits, less vigorous recreational activities, and higher BMI and SBP. In addition, sCr, UA, TG, TC, ALP, and serum phosphorus levels were significantly different between the three groups. Interestingly, the BMD of the total hip, femoral neck, and lumbar spine tended to be significantly higher in patients with diabetes than in prediabetes and NGR subjects.

**Table 1:** Weighted characteristics of US adults ≥ 40 years old with NGR, prediabetes and diabetes, 2005-2018.

	NGR (n=5016)	Prediabetes (n=5730)	Diabetes (n=3864)	*P*
Age (years)	54.57 ± 10.71	59.35 ± 11.40	61.80 ± 11.34	<0.001
Age group				
40-59 years (%)	71.2	53.8	43.7	<0.001
≥60 years (%)	28.8	46.2	56.3	
Male (%)	44.9	49.4	50.8	<0.001
Race/Ethnicity				
Non- Hispanic white (%)	79.5	71.8	65.7	<0.001
Non- Hispanic black (%)	7.2	10.7	12.9	
Mexican American (%)	4.9	6.3	8.5	
Other races	8.5	11.3	12.8	
Education level				
Under high school (%)	12.3	17.9	23.3	<0.001
High school or equivalent (%)	23.2	26.2	26.5	
Above high school (%)	64.5	55.9	50.3	
Marital status				
Married/Living with partner (%)	70.9	68.2	66.3	<0.001
Widowed/Divorced/Separated (%)	22.9	25.4	28.0	
Never married (%)	6.2	6.5	5.7	
PIR				
≤1 (%)	9.0	10.7	12.9	<0.001
1 < to≤3 (%)	28.3	35.5	40.6	
> 3 (%)	62.8	53.8	46.5	
Smoked ≥100 cigarettes in life (%)	46.0	49.6	49.8	0.004
Vigorous recreational activities (%)	23.7	15.3	8.3	<0.001
BMI (kg/m^2^)	27.30 ± 5.34	29.15 ± 5.83	31.50 ± 6.35	<0.001
SBP (mmHg)	123.09 ± 17.56	128.05 ± 18.39	131.66 ± 19.39	<0.001
DBP (mmHg)	72.90 ± 11.56	72.04 ± 12.97	69.38 ± 15.07	<0.001
HbA1c (%)	5.29 ± 0.25	5.67 ± 0.34	6.92 ± 1.53	<0.001
FPG (mmol/L)	4.88 ± 0.43	5.50 ± 0.62	8.15 ± 3.51	<0.001
2h-PG (mmol/L)	5.38 ± 1.21	7.20 ± 1.90	12.81 ± 3.83	<0.001
sCr (umol/L)	79.12 ± 29.81	81.81 ± 25.96	86.43 ± 47.16	<0.001
UA (umol/L)	311.47 ± 78.52	335.10 ± 81.11	340.60 ± 92.28	<0.001
TG (mmol/L)	1.60 ± 1.20	1.84 ± 1.33	2.28 ± 2.19	<0.001
TC (mmol/L)	5.31 ± 1.02	5.25 ± 1.09	4.88 ± 1.24	<0.001
ALP (IU/L)	67.50 ± 21.90	71.80 ± 23.81	74.70 ± 26.81	<0.001
Calcium (mmol/L)	2.36 ± 0.09	2.36 ± 0.09	2.36 ± 0.10	0.104
Phosphorus (mmol/L)	1.21 ± 0.17	1.21 ± 0.18	1.20 ± 0.19	<0.001
Total Hip-BMD (g/cm^2^)	0.933 ± 0.151	0.944 ± 0.159	0.966 ± 0.167	<0.001
Femoral Neck-BMD (g/cm^2^)	0.782 ± 0.136	0.784 ± 0.142	0.799 ± 0.153	<0.001
Lumbar Spine-BMD (g/cm^2^)	1.015 ± 0.148	1.017 ± 0.161	1.045 ± 0.165	<0.001

Mean ± SD for continuous variables; proportion for categorical variables.

NGR, normal glucose regulation; PIR, ratio of family income to poverty; BMI, body mass index; SBP, systolic blood pressure; DBP, diastolic blood pressure; HbA1c, hemoglobin A1c; FPG, fasting plasma glucose; 2h-PG, 2-h plasma glucose; sCr, serum creatinine; UA, uric acid; TG, triglyceride; TC, total cholesterol; ALP, alkaline phosphatase; BMD, bone mass density.

### Association of Glucose Status and BMD

We found a positive trend for glucose status and BMD in the total hip, femoral neck, and lumbar spine (all *P*-trend <0.05) after the adjustment of three models (model 3: β=0.011, 95% CI: 0.004–0.018 for prediabetes; β=0.022, 95% CI: 0.012–0.031 for diabetes in the total hip; β=0.007, 95% CI: 0.001–0.015 for prediabetes; β=0.015, 95% CI: 0.006–0.025 for diabetes in the femoral neck; β=0.002, 95% CI: –0.008–0.012 for prediabetes; β=0.020, 95% CI: 0.007–0.033 for diabetes in the lumbar spine). After stratification by sex, an interesting phenomenon was that men aged ≥ 40 years had no significant association between lumbar BMD and glucose status after multivariate adjustment (*P*=0.149), whereas the trend remained stable in all skeletal sites in the corresponding women (**Table 2**).

**Table 2:** Associations between glucometabolic status and BMD values .

		Total Hip-BMD			Femoral Neck-BMD			Lumbar Spine-BMD		
		β (95% CI) *P-*value			β (95% CI) *P-*value			β (95% CI) *P-*value		
		Model 1	Model 2	Model 3	Model 1	Model 2	Model 3	Model 1	Model 2	Model 3
Total	NGR	Reference	Reference	Reference	Reference	Reference	Reference	Reference	Reference	Reference
	Prediabetes	0.011(0.003,0.018), 0.005	0.027(0.021,0.034), <0.001	0.011(0.004,0.018), 0.002	0.001(-0.006,0.008), 0.714	0.022(0.016,0.028), <0.001	0.007(0.001,0.015), 0.030	0.002(-0.007,0.011), 0.652	0.013(0.004,0.021), 0.003	0.002(-0.008,0.012), 0.689
	Diabetes	0.033(0.024,0.042), <0.001	0.059(0.050,0.067), <0.001	0.022(0.012,0.031), <0.001	0.017(0.008,0.025), <0.001	0.050(0.042,0.058), <0.001	0.015(0.006,0.025), 0.001	0.030(0.019,0.040), <0.001	0.049(0.039,0.060), <0.001	0.020(0.007,0.033), 0.002
	*P*-trend	<0.001	<0.001	<0.001	<0.001	<0.001	0.001	<0.001	<0.001	0.006
Male	NGR	Reference	Reference	Reference	Reference	Reference	Reference	Reference	Reference	Reference
	Prediabetes	0.013(0.003,0.023), 0.012	0.026(0.016,0.037), <0.001	0.010(-0.000,0.021), 0.059	0.006(-0.004,0.015), 0.228	0.021(0.012,0.031), <0.001	0.007(-0.003,0.018), 0.172	0.002(-0.007,0.011), 0.652	0.013(-0.000,0.026), 0.053	0.001(-0.014,0.017), 0.881
	Diabetes	0.024(0.012,0.036), <0.001	0.048(0.036,0.060), <0.001	0.022(0.009,0.035), 0.001	0.011(-0.000,0.022), 0.053	0.040(0.029,0.051), <0.001	0.013(0.001,0.026), 0.040	0.033(0.018,0.048), <0.001	0.035(0.019,0.051), <0.001	0.016(-0.003,0.035), 0.100
	*P*-trend	<0.001	<0.001	0.001	0.048	<0.001	0.035	<0.001	<0.001	0.149
Female	NGR	Reference	Reference	Reference	Reference	Reference	Reference	Reference	Reference	Reference
	Prediabetes	0.012(-0.001,0.024), 0.074	0.031(0.022,0.040), <0.001	0.014(0.005,0.023), 0.003	-0.010(-0.019,-0.001), 0.029	0.024(0.016,0.033), <0.001	0.009(0.000,0.019), 0.046	-0.011(-0.023,0.001), 0.067	0.017(0.005,0.028), 0.005	0.005(-0.008,0.018), 0.465
	Diabetes	0.024(0.011,0.037), <0.001	0.069(0.057,0.082), <0.001	0.022(0.009,0.035), 0.001	0.013(0.000,0.025), 0.049	0.060(0.048,0.071), <0.001	0.018(0.005,0.031), 0.008	0.022(0.007,0.036), 0.003	0.060(0.046,0.074), <0.001	0.021(0.004,0.037), 0.016
	*P*-trend	0.002	<0.001	<0.001	0.261	<0.001	0.005	0.060	<0.001	0.026

Model 1 was unadjusted.

Model 2 was adjusted for age, gender and race.

Model 3 was adjusted for age, gender, race, BMI, education level, marital status, ratio of family income to poverty, smoked at least 100 cigarettes in life, vigorous recreational activities, ALP, total calcium, uric acid, creatinine and cholesterol.

### Association of FPG and BMD

As shown in **Table 3**, after adjusting for potential confounding factors, FPG was found to have a significant association with BMD of the total hip, femoral neck, and lumbar spine in all participants (β=0.003, 95% CI: 0.002–0.005 in the total hip; β=0.003, 95% CI: 0.001–0.004 in the femoral hip; β=0.004, 95% CI: 0.002–0.006 in the lumbar spine). The same conclusion was confirmed in the subgroup of women in the unadjusted, mildly adjusted, and fully adjusted models. After adjustment for age, sex, and race, a positive relationship was found between FPG and femoral neck BMD in the male subgroup (*P <*0.001), while further adjustment for BMI, other socio-demographics, and biochemical indices attenuated its significance (*P*=0.110).

**Table 3:** Associations between fasting plasma glucose and BMD values.

		Model 1 β (95% CI) *P-*value	Model 2 β (95% CI) *P-*value	Model 3 β (95% CI) *P-*value
Total Hip-BMD	Total	0.007 (0.005,0.008), <0.001	0.007 (0.005,0.008), <0.001	0.003 (0.002,0.005), <0.001
	Male	0.003 (0.001,0.005), 0.002	0.005 (0.003,0.007), <0.001	0.003 (0.001,0.005), 0.008
	Female	0.005 (0.003,0.008), <0.001	0.010 (0.007,0.012), <0.001	0.005 (0.002,0.007), 0.001
Femoral Neck-BMD	Total	0.004 (0.003,0.006), <0.001	0.006 (0.004,0.007), <0.001	0.003 (0.001,0.004), 0.003
	Male	0.002 (0.000,0.004), 0.046	0.004 (0.002,0.006), <0.001	0.002 (-0.000,0.004), 0.110
	Female	0.004 (0.001,0.006), 0.002	0.008 (0.006,0.010), <0.001	0.004 (0.001,0.006), 0.005
Lumbar Spine-BMD	Total	0.005 (0.003,0.006), <0.001	0.006 (0.004,0.008), <0.001	0.004 (0.002,0.006), <0.001
	Male	0.003 (0.001,0.005), 0.004	0.004 (0.002,0.006), 0.001	0.003 (0.000,0.006), 0.020
	Female	0.005 (0.002,0.007), <0.001	0.008 (0.006,0.010), <0.001	0.004 (0.002,0.007), <0.001

Model 1 was unadjusted.

Model 2 was adjusted for age, gender and race.

Model 3 was adjusted for age, gender, race, BMI, education level, marital status, ratio of family income to poverty, smoked at least 100 cigarettes in life, vigorous recreational activities, ALP, total calcium, uric acid, creatinine and cholesterol.

### Association of HbA1c and BMD

After adjustment for multivariate factors, there was enough statistical evidence to show a positive association between HbA1c and BMD in all skeletal sites for the whole cohort (β=0.010, 95% CI: 0.007–0.014 in total hip; β=0.008, 95% CI: 0.005–0.012 in femoral hip; β=0.011, 95% CI: 0.006–0.015 in the lumbar spine) and both sexes (**Table 4**).

**Table 4:** Associations between glycohemoglobin and BMD values.

		Model 1 β (95% CI) *P-*value	Model 2 β (95% CI) *P-*value	Model 3 β (95% CI) *P-*value
Total Hip-BMD	Total	0.013 (0.009,0.016), <0.001	0.019 (0.016,0.022), <0.001	0.010 (0.007,0.014), <0.001
	Male	0.009 (0.005,0.013), <0.001	0.014 (0.010,0.018), <0.001	0.009 (0.004,0.014), <0.001
	Female	0.010 (0.005,0.015), <0.001	0.025 (0.020,0.029), <0.001	0.012 (0.007,0.018), <0.001
Femoral Neck-BMD	Total	0.008 (0.005,0.011), <0.001	0.017 (0.014,0.019), <0.001	0.008 (0.005,0.012), <0.001
	Male	0.005 (0.001,0.009), 0.007	0.012 (0.008,0.016), <0.001	0.006 (0.002,0.011), 0.006
	Female	0.007 (0.002,0.011), 0.003	0.022 (0.018,0.027), <0.001	0.011 (0.007,0.017), <0.001
Lumbar Spine-BMD	Total	0.002 (0.006,0.013), <0.001	0.015 (0.011,0.019), <0.001	0.011 (0.006,0.015), <0.001
	Male	0.010 (0.005,0.015), <0.001	0.011 (0.005,0.016), <0.001	0.010 (0.003,0.016), 0.003
	Female	0.008 (0.002,0.013), 0.005	0.019 (0.014,0.024), <0.001	0.011 (0.005,0.016), <0.001

Model 1 was unadjusted.

Model 2 was adjusted for age, gender and race.

Model 3 was adjusted for age, gender, race, BMI, education level, marital status, ratio of family income to poverty, smoked at least 100 cigarettes in life, vigorous recreational activities, ALP, total calcium, uric acid, creatinine and cholesterol.

## Discussion

In the current study, after adjusting for multiple potential confounders, we reported an increasing trend of BMD at the total hip, femoral neck, and lumbar spine with glucometabolic conditions from NGR and prediabetes to diabetes in US adults ≥40 years. This trend was more prominent in women than that in men. Fasting blood glucose and HbA1c levels were also positively related to BMD. To the best of our knowledge, this is the first study to evaluate BMD values in US adults with normal, mild, and overt elevated plasma glucose levels with nationally representative data.

There is a paucity of research on the link between diabetes and BMD values. Some studies have reported that patients with diabetes have decreased BMDs compared to NGR subjects (21, 22), while the majority suggested an average or even higher BMD value (23, 24). Furthermore, few studies have investigated the association between prediabetes and BMD with inconsistent results. A Polish study enrolled 142 subjects (84 men with prediabetes and 58 control men) and found that men with prediabetes had decreased BMD values at the lumbar spine and total body but had a similar BMD at the femoral neck (15). A Chinese study including 173 postmenopausal women with NGR and 73 with IGT reported that the BMD values of IGT were not significantly different from those of NGR (16). In contrast, a study evaluated the BMD dataset from the Korea National Health and Nutrition Examination Survey (KNHANES) and further demonstrated that men with prediabetes and diabetes had increased BMD at all measured sites compared with the control group (14). However, there were comparable BMD values between prediabetes and diabetes in the Korean study, which was different from the findings of our study. In our study, patients with prediabetes and diabetes were observed to have increased BMD, even after adjusting for sex, age, race, BMI, other socio-demographics, and biochemical confounders. Some reasons may explain the diverse results: study design, sample size, examination technology and sites of BMD, and racial differences in BMD and osteoporosis.

The relationship between HbA1c, hyperglycemia, and BMD had conflicting results. A study from Japan (25) indicated that patients with type 2 diabetes (T2D) had a negative correlation between BMD and mean HbA1c at the distal radius in both sexes and at the femoral neck in women. In contrast, a retrospective cross-sectional study (24) found that the prevalence of osteoporosis significantly decreased with increasing blood glucose and HbA1c levels. Our study also demonstrated that FPG and HbA1c levels positively correlate with BMD. However, FPG and HbA1c levels were single measurements at the time of examination and could not reflect the mean blood glucose levels. Averaged HbA1c levels over the entire course of the disease are recommended in future studies.

Our study focused on middle-aged and elderly US individuals and found an increasing trend in BMD in patients with prediabetes and diabetes. However, these results were not in line with the findings in youth with diabetes and prediabetes. An NHANES study of 2005–2006 (26) enrolled youth aged 12-20 years with prediabetes and NGR and demonstrated that children with prediabetes have higher BMD, while adolescents and young adults with prediabetes tended to have lower BMD, suggesting a different biological effect of diabetes progression on bone health during the growing period. A Korean study (27) found that obese children and adolescents with T2D had significantly lower BMD Z-scores at the femoral neck compared with age-, sex-, and BMI-matched controls. A meta-analysis (28) also indicated that children and adolescents with type 1 diabetes (T1D) had lower BMD than controls. Hence, peak bone mass in youth with prediabetes and diabetes might be influenced by the deterioration of bone mineral accruals.

Accumulating evidence from cohort studies and meta-analyses has shown that diabetes is associated with an elevated risk of fragility fractures (29, 30), despite normal or higher BMD values (31). Although DXA is the gold standard for examining bone quality in clinical practice, it may underestimate the risk of fractures in patients with diabetes. A prospective study with a mean follow-up of 12.6 Â years indicated that for a similar hip fracture risk, patients with diabetes had a higher T-score than those without diabetes, and the difference in T-score was 0.59 (95% CI: 0.31–0.87) for women and 0.38 (95% CI, 0.09–0.66) for men (32). In elderly patients with diabetes, a substantially increased risk of falls due to poor balance, peripheral neuropathy, history of coronary heart disease, and arthritis may contribute to a higher risk of fracture (33). Oral anti-diabetic agents and insulin also exert their effects on bone strength (8). Other psychosocial factors, including cognitive impairment, should be considered in fracture risk assessments (34). The results of our study indicate that the mean age of the diabetes group was higher than that of the NGR and prediabetes groups, with paradoxically higher BMD. BMI is considered a protective factor against osteoporosis (35). Therefore, several confounders were adjusted in our study to investigate the independent effect of glucometabolic conditions on BMD. However, a cohort study suggested that the impact of diabetes on fractures was increased in the BMD-added prediction model (36). Hence, more comprehensive studies are needed to evaluate whether BMD is an independent predictor of fragility fractures in individuals with diabetes.

In the current study, we analyzed data from the NHANES, which is unique in collecting demographic, health, and nutritional information. The NHANES sample represents the national US civilian population. In addition, the interview and examination processes were under high-quality control to ensure the reliability of the data. This is the key strength of this study. Furthermore, diabetes and prediabetes diagnosis were based on self-reported diagnosis and on the results of OGTT and HbA1c, increasing the diagnostic accuracy of glucose metabolism. However, this study had several unavoidable limitations. First, because of the cross-sectional nature of the NHANES, it is difficult to conclude on the cause-effect relationship. Second, anti-diabetic medicine was not included in the diabetes group, which may negatively or positively influence BMD. Third, although we examined the changes in socio-demographics, BMI, and biochemical indexes, other potential residual confounders, such as vitamin D and calcium supplementation, use of glucocorticoids may still need to be considered. Therefore, more prospective studies are required to investigate bone metabolism at different glucose levels.

In conclusion, based on nationally representative data extracted from the NHANENS database from 2005 to 2018, we found a positive association between glucometabolic conditions and BMD values in US adults. To better understand the mechanisms underlying bone metabolism in individuals with prediabetes and diabetes, more longitudinal research is needed to explain BMD changes from normoglycemia to prediabetes and diabetes.

## Data Availability Statement

The original contributions presented in the study are included in the article/supplementary material. Further inquiries can be directed to the corresponding authors.

## Ethics Statement

The studies involving human participants were reviewed and approved by NHANES. The patients/participants provided their written informed consent to participate in this study.

## Author Contributions

JY extracted data from NHANES database, analyzed the data, wrote the manuscript. PJ and J-BZ conceived the idea for the study and edited the manuscript. All authors read and approved the final manuscript. All authors contributed to the article and approved the submitted version.

## Funding

This work was supported by the National Natural Science Foundation of China (grant numbers 82070851, 81870556), the Beijing Municipal Administration of Hospitals’ Youth Program (grant number QML20170204), and Excellent Talents in the Dongcheng District of Beijing.

## Conflict of Interest

The authors declare that the research was conducted in the absence of any commercial or financial relationships that could be construed as a potential conflict of interest.

## Publisher’s Note

All claims expressed in this article are solely those of the authors and do not necessarily represent those of their affiliated organizations, or those of the publisher, the editors and the reviewers. Any product that may be evaluated in this article, or claim that may be made by its manufacturer, is not guaranteed or endorsed by the publisher.
